# A protocol for an experimental investigation of the effects of pain-related interpretation bias modification on interpretation of ambiguous somatosensory and linguistic stimuli in healthy individuals

**DOI:** 10.1371/journal.pone.0318296

**Published:** 2025-04-07

**Authors:** Philippa Broadbent, Christina Liossi, Daniel Eric Schoth

**Affiliations:** School of Psychology, University of Southampton, Highfield, Southampton, United Kingdom; University of Oxford, AUSTRALIA

## Abstract

Researchers are increasingly exploring the existence and potential implications of pain-related cognitive biases in clinical and non-clinical populations, using a range of paradigms and stimuli to test theoretical predictions and refine models. One avenue of investigation explores the implications of biased pain-related interpretations in pain-free individuals, which may shed light on processes of transition from a pain-free state to acute pain, and acute pain to chronic pain. The primary aim of the main study detailed in this protocol is to investigate the effects of pain-related cognitive bias modification for interpretation (CBM-I) on the interpretation of ambiguous somatosensory stimuli. When deciding the point at which to report their level of pain threshold and tolerance, participants must by necessity interpret somatosensory sensations. Sensations around the pain threshold are likely to be ambiguously painful, sometimes interpreted as painful and sometimes not, which has been proposed as a zone of uncertainty. A pilot study was first conducted to test whether the proposed experimental design is feasible and appropriate, and to ascertain study effect sizes. Eighteen healthy participants were randomised to complete either benign CBM-I (trained towards neutral interpretations of ambiguous scenarios; n =  9) or pain CBM-I (trained towards pain-related interpretations of ambiguous scenarios; n =  9). The Ambiguous Scenarios Task was used to train interpretation biases, and a novel Interpretation of Somatosensory Sensations Assessment was used to explore interpretation of ambiguous somatosensory sensations around the individual’s pain threshold. Participants receiving pain CBM-I, compared to those receiving benign CBM-I, had higher pain-related interpretation bias scores on the test phase of the Ambiguous Scenarios Task with a large effect size, and showed higher intensity and unpleasantness ratings on the Interpretation of Somatosensory Sensations Assessment typically with medium to large effect sizes. These paradigms are suitable for a full-scale investigation. Modifications made to the experimental design based on insights from the pilot study are discussed. This research extends beyond pain patient populations to researchers and clinicians working with other conditions, especially anxiety and mood disorders where patients may misinterpret or catastrophize ambiguous bodily sensations.

## Introduction

Pain-related interpretation bias can be defined as the tendency to interpret ambiguous information in a pain-related manner. The fear-avoidance model of pain posits that interpretation of ambiguous pain information as threatening contributes to the maintenance of pain and disability via increases in fear, catastrophising and subsequent avoidance of activity, which promotes pain [1,2]. In recent years two lines of investigation have explored the existence and implications of pain-related interpretation biases. The first has focused on chronic pain, with a meta-analysis from our research group showing patients with chronic pain compared to healthy controls demonstrate significantly greater interpretation bias favouring pain- or illness-related meanings of symbolic linguistic and pictorial stimuli across four paradigms (word stem completion task, homographic response task, homophone task, and incidental learning task) [3]. Recent studies have provided further evidence that individuals with chronic pain show significantly greater interpretation biases for pain- or illness-related written scenarios compared to healthy individuals (e.g., [4,5]).

Studies have also explored the implications of cognitive bias modification for interpretation (CBM-I). One study recruiting participants with mixed chronic pain conditions found those trained towards objective/benign interpretations of ambiguous pictures, compared to controls receiving no training, showed significant decreases in interpretation biases for ambiguous words, lower levels of negative emotions in response to pain-related pictures and less dwell time bias on novel affective pain words [6]. Sharpe and colleagues [7] conducted the first online study using remotely delivered CBM-I for people with mixed chronic pain conditions. Participants in the intervention group were trained to interpret written ambiguous scenarios as benign, whereas those in the control group completed a task which did not require any emotional interpretation or any resolution of ambiguity. Participants in the intervention group showed statistically significant greater improvements on primary outcomes of pain interference and pain intensity compared to those in the control group, with the authors suggesting clinically meaningful outcomes in approximately 15–20% of those receiving CBM-I.

The second line of investigation has explored potential implications of biased pain-related interpretations in healthy, pain-free individuals, which may shed light on processes of transition from a pain-free state to acute pain, and acute pain to chronic pain. Vancleef and colleagues [8] found biased interpretations favouring health-threat were significantly associated with increased pain-related anxiety and lower tolerance for heat pain in healthy participants. The researchers concluded that biased interpretations may serve as a latent vulnerability mechanism in the development of chronic pain. Furthermore, recent research with patients with acute pain found health-related interpretation bias was associated with increased pain-related anxiety, which predicted pain severity and pain interference at three months [9]. Despite the important implications of these findings, few studies have explored CBM-I for pain in healthy individuals. One study training healthy participants towards threatening or nonthreatening interpretations of pain revealed those in the former group compared to the latter group showed greater pain-related interpretations of ambiguous scenarios and greater hesitance (avoidance) before a cold-pressor task [10]. Another study [11] found significant reductions in pain-related interpretation biases, lower levels of negative emotions to pain-related images and higher pain thresholds (as assessed via the cold pressor task) in healthy participants trained towards benign/objective interpretations of ambiguous stimuli compared to participants in a control condition who received no training. Furthermore, the effect of bias modification on pain threshold was mediated by changes in interpretation bias.

The results of these two studies suggest that patterns of interpretation bias can be modified in healthy individuals using the Ambiguous Scenarios Task, which can subsequently impact performance on the cold pressor task. When deciding the point at which to report their level of pain threshold and tolerance, participants must by necessity interpret somatosensory sensations. Sensations around the pain threshold are likely to be ambiguously painful, sometimes interpreted as painful and sometimes as non-painful to the same individual, which has been proposed as a zone of uncertainty [12]. It is currently unclear whether CBM-I has a greater influence on interpretation of stimuli under or over the pain threshold, although the cold pressor task as used in former CBM-I research [10,11] does not allow for investigation of this question, which requires precise and fast administration of stimuli around each participant’s individual pain threshold. Experimentally delivered thermal stimuli administered via computerised thermal neurosensory analyser allows for testing of such hypotheses, as all aspects of the thermal stimulus presentation can be precisely controlled, including target temperature, duration of the thermal stimulus, and stimulation rates (i.e., the speed with which the target temperature is reached).

The primary aim of the main study detailed in this protocol is to investigate the effect of pain-related CBM-I on the interpretation of ambiguous somatosensory stimuli explored via a novel paradigm developed specifically for this investigation. Healthy, pain-free participants will be recruited as they are less likely to have pain-related cognitive biases than individuals with chronic pain [3,13]. This makes it possible to train such biases toward pain-related interpretations, as demonstrated in previous research [10]. To inform the development of this protocol, a pilot study was conducted to test the experimental methods and procedure and to establish study effect sizes. This was conducted in line with the NIHR, which defines pilot studies as ‘a smaller version of the main study used to test whether the components of the main study can all work together’[14] and which focuses on the processes to be followed in the main study to ensure they will run smoothly [15]. The methods and results of this pilot study will be presented first, followed by a discussion of insights gained and amendments made to the final protocol for the main experimental study.

## Pilot study aims

The overarching aims of the pilot study were to test the feasibility of the experimental methods and procedure implemented and to establish study effect sizes, informing the final experimental design and necessary sample size in the main study. A novel experimental paradigm was developed for this study, i.e., the Interpretation of Somatosensory Sensations Assessment, with participant engagement, task comprehension, and the paradigm’s suitability for future research was evaluated. The primary goal of the pilot study was to investigate the effect of pain-related CBM-I on the interpretation of ambiguous somatosensory stimuli in healthy, pain-free individuals. The secondary goal was to investigate the effects of CBM-I on directly and indirectly measured interpretation of ambiguous, pain-related language. Direct measures are based on the participant’s response (e.g., verbally stating or writing their interpretation), while indirect measures are based on performance behaviour (e.g., response latencies) [16,17]. Previous research has found direct measures of interpretation bias are not significantly associated with indirect measures [18,19], although this has been tested by few studies. Both goals were in relation to the overarching aim of this pilot study to provide data and information informing the development of a final protocol for a full-scale study. This pilot study used a between-groups design with two groups of healthy participants, one randomised to complete benign CBM-I (trained towards neutral interpretations of ambiguous scenarios) and the other completing pain CBM-I (trained towards pain-related interpretations of ambiguous scenarios).

## Pilot study methods

### Participants

Approval was obtained from the University of Southampton Research Ethics Committee (ERGO ID: 54360). Inclusion criteria were (i) aged 18–50 years; (ii) normal or corrected to normal vision; and (iii) fluency in the English language. Exclusion criteria were: (i) diagnosed with any type of chronic pain or psychiatric condition; (ii) currently experiencing pain; or (iii) any known reading difficulties. Participants were recruited using posters around the University of Southampton campus, social media, word-of-mouth, and the website ‘eFolio’ which is used to recruit BSc Psychology students at the University of Southampton. Participants were compensated £12 (£6 per hour). BSc Psychology students at the University of Southampton could instead receive partial course credit for participating. Participants provided informed written consent, witnessed by the researcher PB. The recruitment period was from 21/02/20–23/03/20.

### Self-report measures

Self-report measures of constructs associated with cognitive biases and/or experimental pain thresholds were administered in a newly randomised order (conducted via Random.org) for each participant. Anxiety was measured via the State-Trait Anxiety Inventory (STAI) [20], which has excellent internal consistency (Cronbach’s alpha .91 and .89 for state and trait subscales respectively) [21]. A review of seven articles found acceptable (.70) and excellent (.89) test-retest reliability coefficients for state and trait subscales respectively [21]. Cognitive biases in attention and interpretation have been commonly implicated in cognitive models of anxiety [22]. Anxiety sensitivity was assessed via the Anxiety Sensitivity Index-3 (ASI-III) [23], which has good to excellent internal consistency (Cronbach’s alpha .94 for total score, .86, .90 and .80 for physical concerns, cognitive concerns and social concerns subscales respectively [24]). Research has shown adequate to excellent test-retest reliability (e.g., *r = *.70,.60 and .82 for physical concerns, cognitive concerns and social concerns subscales respectively [25]). Anxiety sensitivity has been associated with higher interpretation bias and greater pain [26] and heighted attentional biases towards health-threat [27] in pain-free individuals. Depression was assessed via the Beck Depression Inventory-II (BDI-II) [28] which has excellent internal consistency (Cronbach’s alpha: .90 [29]) and test-retest reliability (*r* = .93 [28]). Although debate exists within the literature, several reviews have shown depression to be associated with cognitive biases for negative information [30,31].

Awareness of visceral and somatic sensations was assessed via the Somatosensory Amplification Scale (SSAS; [32]), which has acceptable internal consistency (Cronbach’s alpha = .70) and good test-retest reliability (*r* = .79) [32]. Research has shown heightened somatosensory amplification is associated with decreased pain thresholds in healthy individuals [33]. Fear of pain was assessed via the Fear of Pain Questionnaire – III (FOP-III; [34]), which possesses good internal consistency (Cronbach’s alpha total score = .92; severe = .88; minor = .87, medical = .87) and test-retest reliability (Cronbach’s alpha total score = .74; severe = .69; minor = .73, medical = .76) [34]. Several studies have found heightened fear of pain to be associated with increased attentional biases for pain-related information [35,36]. Pain catastrophising was assessed via the Pain Catastrophising Scale (PCS; [37]), which has acceptable to excellent internal consistency (Cronbach’s alpha .87 (rumination subscale), .66 (magnification subscale), .78 (helplessness subscale), .87 (overall score) [37]) and good test-retest reliability ((Spearman ρ =  0.88; [38]). Research has shown higher levels of pain-related catastrophizing to be related to higher suprathreshold pain ratings and greater temporal summation of thermal pain [39]. Further to these measures, the Edinburgh Handedness Questionnaire – Short Form [40] was administered to confirm hand dominance for quantitative sensory testing, which has excellent reliability ((alpha =  93) [40]), along with a demographic questionnaire assessing sex, date of birth, occupation, level of education, and chronic pain among close relatives. The questionnaires took approximately 20 minutes to complete.

### Stimuli and experimental paradigms

#### Stimuli.

The Ambiguous Scenarios Task used stimuli developed by Jones and Sharpe [10], with adjustments made to account for differences between Australian and British English. Specifically, ‘capsicum’ was changed to ‘pepper’, ‘sun-baking’ to ‘sun-bathing’, and ‘solarium’ to ‘sunbed’ (S1 File). Ambiguous pain-related words and neutral homographs were used in the Sentence Generation Task. Pain homographs had sensory-pain and neutral meanings, and were derived from the McGill Pain Questionnaire [41]. Neutral homographs had multiple neutral meanings. The neutral and pain homographs were matched on length and written frequency according to the MRC Psycholinguisic Database [42] and on number of strong associations according to the University of Florida Free Association Norms Database [43]. For the training phase of the Incidental Learning Task, unambiguous sensory-pain words and neutral homographs were used (the latter were the same as those used in the sentence generation task; although they have multiple meanings, none of these were pain-related and so were suitable as neutral training stimuli). Ambiguous words with sensory-pain and neutral meanings were used in the testing phase of the Incidental Learning Task. These stimuli have previously been used in attention, interpretation and memory bias research from our lab [44,45], and are shown in Table 1.

**Table 1:** Sensory-pain and neutral words used in the sentence generation task and the incidental learning task.

Ambiguous words with sensory-pain and neutral meanings	Neutral homographs with multiple meanings	Unambiguous pain words
Tension	Setting	Aches
Pounding	Boarding	Harmful
Pressing	Inclined	Painful
Splitting	Promotion	Hurting
Piercing	Animated	Pinching
Drilling	Compound	Stinging
Tight	Grasp	Headache
Pulsing	Banking	Soreness
Squeeze	Trailer	Agonising

#### Ambiguous scenarios task.

CBM-I training was achieved via the Ambiguous Scenarios Task [46] modified for pain research [10]. In the training phase, descriptions of ambiguous scenarios were presented. After reading the description, participants completed the final word from a fragment and then answered a yes/no comprehension question. In the pain modification condition, the final word gave the scenario a pain-related meaning, whereas in the benign modification condition the final word gave the scenario a neutral meaning. An example sentence is: ‘You and your friend are preparing dinner. She cuts the onion and you cut the capsicum. Suddenly her knife slips and cuts into your…’, with ‘c_ps_c_m’ (capsicum) and ‘f_ng_r’ (finger) as non-pain and pain interpretations respectively. Thirty descriptions were presented in a random order, with each then presented a second time again in new random order. The 60 descriptions and questions were presented as one block. In this phase, participants in the pain modification group learnt ambiguous scenarios have painful endings whereas participants in the benign modification group learnt ambiguous scenarios have benign endings.

The test phase serves as a direct measure of interpretation bias, investigating whether participants interpret new ambiguous descriptions as pain-related or benign. If the modification is successful, participants in the pain modification group should make more pain-related interpretations than the benign modification group. In the first part of the test phase, 10 ambiguous descriptions were presented, each with a meaningful title. Participants were asked to complete the fragment of the final word, but this word will not disambiguate the situation. Participants were also asked a comprehension question about each description to ensure they understood the meaning. In the second part of the test phase, the title of each scenario was shown, along with four possible endings for the scenario, all of which differ slightly from the original ending. Two endings were pain-related and two were neutral. One was more relevant to the bias modification (i.e., pain-related) the other less relevant (i.e., generally negative). Participants were asked to rate the similarity of the four endings to the original scenario from 1 (very different in meaning) to 4 (very similar in meaning). Participants were instructed that although none of the endings are identical to the original description, any of them could be similar in meaning. Participants were also asked to rate how easily they could imagine themselves as the protagonist in the descriptions from 1 (not at all) to 5 (extremely) as a measure of visual imagery, which has previously been found to enhance the effectiveness of interpretation bias modification [47]. This task took approximately 20 minutes to complete.

#### Sentence generation task.

This is a direct measure of interpretation bias, which has been successfully implemented in previous research from our research group [44,45]. The task included 18 experimental trials featuring nine ambiguous sensory-pain and nine ambiguous neutral stimuli [44]. Trials were presented in a new randomised order for each participant. Each trial began with a fixation cross for 1000ms. A single word replaced the cross, presented in size 40 Times New Roman font which remained on the screen until the end of the trial. Participants read the word and used a keyboard to type a single sentence featuring the word once. Text appeared in size 18 Times New Roman font below the experimental word as the participant typed. Backspace and delete keys were used to correct spelling mistakes or make amendments as necessary, and the F12 key was used submit the response. The next trial began after 120 seconds if no response was submitted. Trials followed one another automatically, with all 18 trials presented in a single block. Two practice trials were initially presented to familiarise participants with the requirements of the task, featuring the words *running* and *dancing*. This task took approximately 15 minutes to complete.

#### Incidental learning task.

This is an indirect measure of interpretation bias. In the learning phase, each trial begins with a central fixation presented for 500ms. Following this, an unambiguous pain or neutral word was presented centrally for 675ms. Following pain words, a dot was presented on one side of the screen (e.g., the left) for 80% of trials and on the other side for 20% of trials. The opposite was the case for neutral words. Participants responded to the location of the dot as quickly as possible using the left and right arrows on the keyboard. The side associated with pain words was counterbalanced between participants. There was an inter-trial interval of either 800ms or 1200ms, varying randomly. The same series of events occurred in the test phase, except an ambiguous pain word was shown in the centre of the screen and the dot had an equal chance of appearing on the left or right. Faster responses to dots on the side previously predicted by pain words indicates pain-related interpretation bias. Each word was presented four times, therefore there were 72 trials in the learning phase and 36 trials in the testing phase. The trials in each phase were presented in a randomised order. Participants completed 10 practice trials with neutral words and had the opportunity to ask questions before the start of the main block of trials. This task took approximately 10 minutes to complete.

### Quantitative sensory testing

#### Pain threshold measurement.

Heat pain thresholds were measured using a Neurosensory Analyser Model Thermal Sensory Analyser-II (TSA-II). The thermode has a contact area of 3 cm^2^ and was attached to the thenar eminence of the participant’s non-dominant hand with a Velcro strap. The thermal stimulus was increase by 1°C/second from its baseline temperature of 32°C until the participant indicated the very first moment they felt the temperature as painful by pressing the left mouse button. The thermal stimulus had upper and lower safety limits of 50°C and 0°C respectively. The thermal stimulus decreased by 8°C/second back to baseline temperature. Three trials were conducted, with a 10-second inter-trial interval. This task took approximately 10 minutes to complete.

#### Interpretation of somatosensory sensations assessment (ISSA).

**Fig 1:**
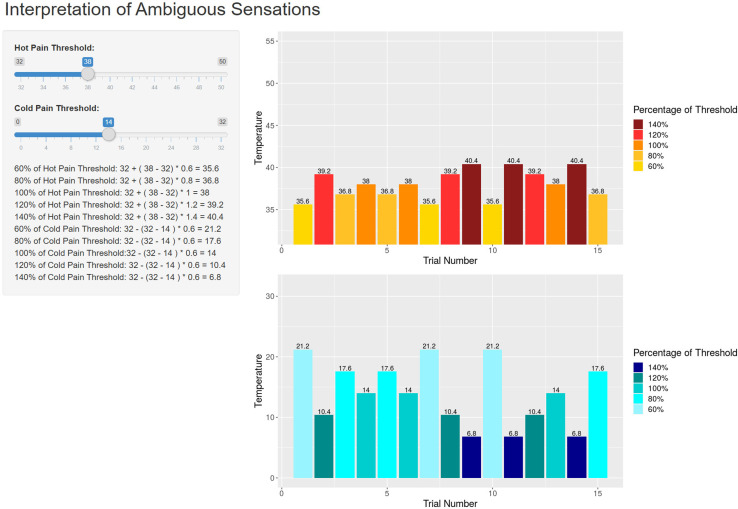
Example of the Shiny app for computing ambiguous pain thresholds. In this example heat and cold pain thresholds of 38C and 14C respectively have been entered on the left, and the ambiguous pain thresholds to enter into the Thermal Sensory Analyser are provided on the right (heat trials above, cold trials below).

This is a novel paradigm developed specifically for this study. Heat stimuli were administered to the non-dominant hand using the TSA-II. Stimuli were presented at 60%, 80%, 100%, 120% and 140% of an individual’s heat pain threshold three times each, in a random order set across participants, which was calculated individually for each participant using an app created in R-Shiny [48] (Fig 1; available at https://phillybroadbent.shinyapps.io/temp_calculations_app/). Percentage threshold temperature values exceeding the safety limits of the TSA-II were capped at 50°C. Each trial began with the stimulus increasing from baseline temperature at a rate of 1°C/second until reaching the target temperature. The target temperature was held for three seconds, before returning to baseline by 8°C/second. At this point, participants rated, on pen and paper visual analogue scales, the intensity, from 0 (no sensation) to 100 (most intense pain you can imagine), and the unpleasantness of the heat stimulus, from 0 (not unpleasant at all) to 100 (extremely unpleasant). The fifteen heat trials were presented consecutively with a 10 second inter-trial interval. This task took approximately 10 minutes to complete.

### Procedure

**Fig 2:**
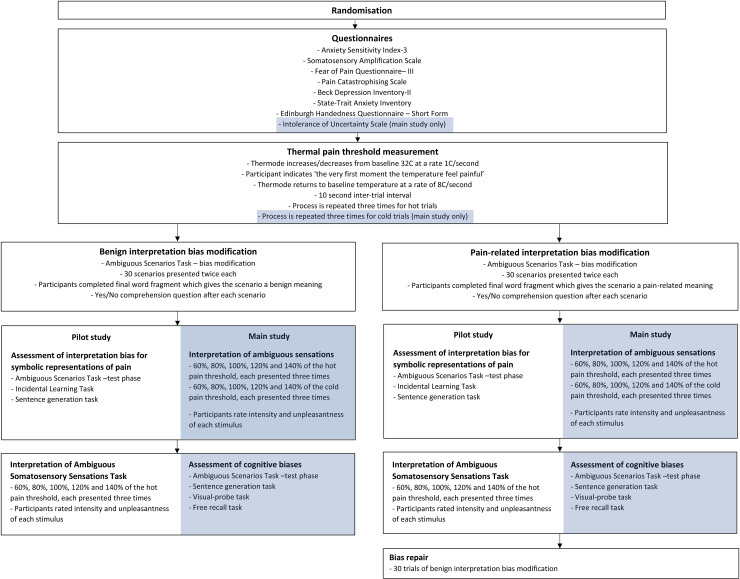
A flowchart showing the experimental procedure for the pilot study and main study exploring the effects of pain-related interpretation bias modification on interpretation of ambiguous somatosensory and linguistic stimuli in healthy individuals (shaded components show main study differences to the pilot study).

The procedure for this study is shown in Fig 2. Participant ID numbers were randomly assigned to the benign or pain modification group before data collection began via Random.org. After having read the information sheet and signed the consent form, participants completed the self-report measures. Following this, participants completed the heat pain-threshold task. Next, participants completed the Ambiguous Scenarios Task aiming to modify their biases towards pain-related or benign interpretations of ambiguous stimuli, according to their randomised group. This was immediately followed by the test phase of the Ambiguous Scenarios Task, then the Incidental Learning Task and the Sentence Generation Task. Participants then completed the ISSA. At the end of the experiment, participants in the pain-related CBM-I group completed 30 trials of the benign modification to ensure that they did not experience lasting effects of pain-related bias modification, before being thanked and debriefed. The entire experimental duration was approximately 90 minutes.

### Data reduction and analytic plan

Data was analysed using RStudio version 1.3.1073 [49] with R version 4.0.2 [50]. As this is a pilot study, descriptive statistics and Cohen’s *d* effect sizes only are reported. Pearson’s correlation coefficients were also computed across all participants between self-report measures with measures of interpretation bias and interpretation of somatosensory sensations using the R package ‘corrplot’ [51]. Self-report measures were scored according to their published instructions. For the Ambiguous Scenarios Task, the proportion of correct comprehension questions was calculated to determine engagement with the task. Mean scores for each category of ending were calculated for each participant. The mean score for benign target endings was be subtracted from the mean score for negative endings to give an index of biased interpretation, as in previous use of the Ambiguous Scenarios Task [52]. A positive interpretation bias index indicates pain-related interpretation bias.


Ambiguousscenariosindex=TN−TB

Where *TN* =  mean score for target negative endings and *TB* =  mean score for target benign endings.

For the Incidental Learning Task, trials with incorrect or no response were excluded (learning phase: 1.39%, testing phase: 1.54%). Histograms and boxplots showed that response times less than 200ms or greater than 800ms were outliers, so these were removed (learning phase: 1.00%, testing phase: 1.08%). Response times more than three standard deviations outside each participant’s mean response time were excluded (learning phase: 1.70%, testing phase: 1.54%). The congruency effect was calculated with data from the learning phase. A positive congruency effect indicates a faster response when the dot appears on the predicted side of the screen, indicating that the contingency has been learnt.


Congruencyeffect=RTi−RTc

Where *RTi* =  mean response time on incongruent trials and *RTc* =  mean response time on congruent trials. The IB index was calculated from response times in the test phase, as in previous research [18,53]. A positive index indicates pain-related interpretation bias.


Interpretationbiasindex=RTn−RTp

Where *RTn* =  mean response time to targets in the location associated with neutral words and *RTp* =  mean response time to targets in the location associated with pain words.

For the Sentence Generation Task, sentences were categorised as pain-related or benign. The definition for pain-related interpretations was: ‘describes a painful experience or a situation usually associated with pain’; the working definition for benign interpretations was: ‘describes an experience or situation unrelated to pain’ [44,54]. The proportion of pain-related sentences out of total sentences generated was calculated, as in previous research [17].

For the ISSA, mean ratings of intensity and unpleasantness were calculated for each stimulus intensity. Ratings were excluded where the temperature required for the percentage of pain threshold could not be reached due to the safety limits of the TSA-II.

## Pilot study results

Data was collected from 18 participants (12 female, 5 male, one did not provide data) with a mean age of 25.58 years (SD =  8.55). Nine participants completed benign modification (seven female, two male) and nine completed pain modification (five female, three male, one did not provide data). Table 2 presents descriptive statistics for the self-report measures completed.

**Table 2:** Descriptive statistics for self-report measures completed in the pilot study (N =  18).

Self-report measure	Benign CBM-I group mean (SD) (n = 9)	Pain CBM-I group mean (SD) (n = 9)
STAI-State	29.44 (4.59)	36.56 (11.37)
STAI-Trait	37.89 (9.83)	41.22 (9.15)
ASI-3 Social	8.11 (3.18)	13.78 (4.94)
ASI-3 Physical	3.44 (3.57)	9.78 (6.5)
ASI-3 Cognitive	1.67 (2.24)	6.44 (3.5)
ASI-3 Total	13.22 (5.63)	30.00 (8.03)
BDI-II	6.33 (4.24)	6.00 (3.64)
SSAS	25.78 (5.33)	29.78 (4.41)
FPQ-III Minor	17.56 (4.82)	20.11 (6.09)
FPQ-III Medical	25.89 (5.16)	25.78 (4.02)
FPQ-III Severe	35.78 (7.66)	38.44 (7.54)
FPQ-III Total	79.22 (12.63)	84.33 (16.53)
PCS Rumination	8.00 (2.00)	8.89 (3.55)
PCS Magnification	3.22 (2.11)	5.56 (2.51)
PCS Helplessness	7.11 (4.23)	12.11 (3.66)
PCS Total	18.33 (7.75)	26.56 (7.97)

ASI =  Anxiety Sensitivity Index; SSAS =  Somatosensory Amplification Scale; FPQ-III =  Fear of Pain Questionnaire-III; PCS =  Pain Catastrophising Scale; BDI-II =  Beck Depression Inventory II; STAI =  State-Trait Anxiety Inventory.

### Task evaluations

For the Ambiguous Scenarios Task, on average 93.70% (SD =  5.07%) of comprehension questions were answered correctly in the learning phase and 97.78% (SD =  6.47%) in the test phase, indicating a high level of engagement. For the Incidental Learning Task, the mean learning congruency effect was -1.63 (SD =  19.78), which indicates responses were slightly faster on incongruent than congruent trials. This suggests that the contingency between word type and dot location was not learnt. For the Interpretation of Ambiguous Sensations Assessment, 120% or 140% of thresholds could not be reached for several participants due to the safety limits of the TSA-II. Specifically, 140% of the pain threshold was higher than 50°C for five participants (3 benign group, 2 pain group) and 120% was higher than 50°C for two participants (1 benign group, 1 pain group).

### Interpretation of ambiguous somatosensory sensations assessment

**Fig 3:**
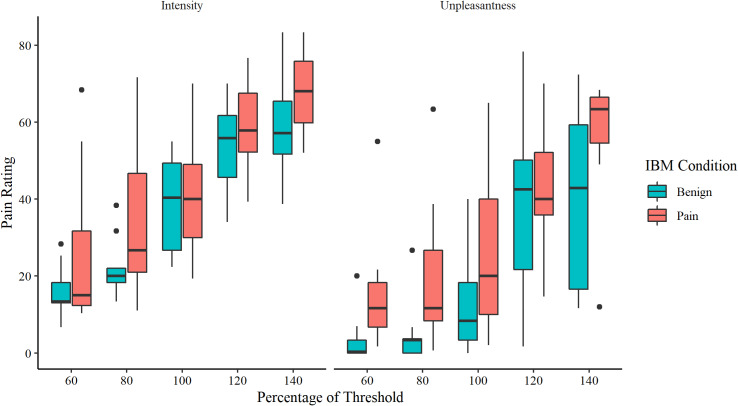
A boxplot showing the intensity and unpleasantness ratings for stimuli at 60% to 140% of the hot pain threshold. The central line indicates the median, the upper and lower ends of the box indicate the upper and lower quartiles of the data. The error bars indicate 1.5 times the interquartile range and dots indicate data points more than 1.5 times above or below the interquartile range beyond either end of the box.

As shown in Table 3, intensity and unpleasantness ratings were higher for the pain modification group than the benign modification group for stimuli at all threshold percentages. Boxplots for intensity and unpleasantness ratings are shown in Fig 3.

**Table 3:** Descriptive statistics and between-group differences with effect sizes for intensity and unpleasantness ratings on the interpretation of ambiguous sensations task (N =  18).

Stimulus Type	Benign modification group mean (SD)	Pain modification group mean (SD)	Cohen’s *d*
Intensity Ratings			
60%	16.30 (7.03)	27.22 (21.08)	0.69
80%	22.22 (7.99)	34.48 (19.62)	0.82
100%	38.44 (11.96)	41.11 (16.31)	0.19
120%	54.29 (12.31)	58.08 (12.76)	0.30
140%	52.6 (10.41)	70.75 (11.27)	1.95
Unpleasantness Ratings			
60%	3.59 (6.58)	15.37 (16.22)	0.95
80%	5.04 (8.41)	20.30 (19.81)	1.00
100%	13.89 (13.62)	25.63 (20.77)	0.67
120%	37.29 (26.05)	44.00 (16.68)	0.31
140%	26.53 (24.22)	61.92 (7.14)	3.02

### Assessments of interpretation biases

**Fig 4:**
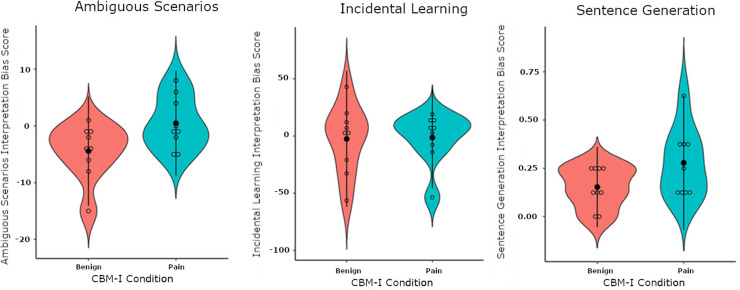
Violin plots showing interpretation bias scores on the Ambiguous Scenarios Task, Incidental Learning Task and Sentence Generation Task grouped by interpretation bias modification condition. The width of the violin plots represents the density of the data at each value, with wider sections indicating higher density. Unfilled dots indicate the scores for each individual participant, the black dot indicates the mean across participants, and the error bars represent ± 2 standard deviations.

Pain-related interpretation bias scores on the Ambiguous Scenarios Task were higher for participants in pain modification group (M =  0.44, SD =  4.61) than participants in the benign modification group (M =  -4.44, SD =  4.82), with a large effect size (Cohen’s *d* =  1.03). The interpretation bias index from the Incidental Learning Task did not differ much between the benign modification group (M =  -2.52, SD =  29.76) and the pain modification group (M =  -1.37, SD =  22.26), with a very small effect size (*d* =  -0.05). For the Sentence Generation Task, the proportion of pain-related sentences generated was higher for the pain modification group (M =  0.28, SD =  0.17) than the benign modification group (M =  0.15, SD =  0.10), with a small effect size (*d* =  -1.02). Violin plots for interpretation bias scores for all three paradigms are shown in Fig 4.

### Correlational analyses

Anxiety sensitivity was positively associated with unpleasantness ratings for stimuli at 60% (*r* = .51) and 80% (*r* = .51) of the pain threshold. State anxiety was positively associated with intensity ratings for stimuli at 80% (*r* = .48), and 100% (*r* = .48), of the pain threshold, as well as with unpleasantness ratings for stimuli at 60% (*r* = .72), 80% (*r* = .75) and 100% (*r* = .77) of the pain threshold. Trait anxiety was positively associated with intensity ratings for stimuli at 140% (*r* = .48) of the pain threshold, and with unpleasantness ratings for stimuli at 60% (*r* = .61), 80% (*r* = .55), 100% (*r* = .59), 120% (*r* = .56), and 140% (*r* = .54) of the pain threshold. Anxiety sensitivity was positively associated with the proportion of pain sentences generated (*r* = .57). Additionally, scores on the test phase of the Ambiguous Scenarios Task and Sentence Generation Task were correlated (*r* = .68).

## Insights for the main experimental study

The results of this pilot study provided important insights for the main investigation. In line with former studies [10,11], the Ambiguous Scenarios Task appears suitable for this line of investigation. Participants showed a high level of engagement with the task, and those allocated to the pain modification group showed higher intensity and unpleasantness ratings on the ISSA than those in the benign modification group, typically with medium to large effect sizes. Furthermore, participants in the pain modification group had higher pain-related interpretation bias scores on the test phase of the Ambiguous Scenarios Task compared to those in the benign modification group, with a large effect size. Taken together, this suggests the Ambiguous Scenarios Task with linguistic stimuli is capable of modifying interpretation biases, which in turn affects intensity and unpleasantness ratings for ambiguous somatosensory sensations. Considering the other cognitive paradigms, little difference in interpretation scores were shown between the two modification groups on the Incidental Learning Task, with the results suggesting a lack of learning across participants. Few former studies have used this paradigm to explore pain-related biases, although these found pain-free individuals can learn the association between stimulus type and dot location [55,56]. While it is unknown why learning did not occur in the present pilot study, these former studies used ambiguous facial expressions as opposed to linguistic stimuli. Considering the Sentence Generation Task, a higher proportion of pain-related sentences was generated by the pain modification group than the benign modification group. Although the effect size was small, this nevertheless implies this task may be more suitable for the current line of investigation using linguistic stimuli than the Incidental Learning Task. During the ISSA, 120% and 140% heat thresholds could not be reached for five and two participants respectively due to TSA-II safety limits of 50°C. This indicates that the paradigm requires modification while importantly adhering to safety limits of the equipment.

Based upon these findings and results from the broader literature, the following modifications have been made to the experimental protocol for the main investigation. First, the primary outcome of interpretation of ambiguous somatosensory sensations will be performed immediately following CBM-I, with assessment of post CBM-I cognitive biases performed after this. This will eliminate the possibility that assessing post-CBM-I cognitive biases reduces the training effect. Second, assessments of pain-related attentional bias and memory bias will also be conducted following CBM-I. Pain-related biases in attention (e.g., [57–68]) and memory (e.g., [45,69–74] have been frequently explored by our research group and others across a range of populations and using a range of paradigms, and their inclusion in the protocol will enable an assessment of the inter-relatedness of different forms of cognitive bias as predicted by theoretical models such as the Threat Interpretation Model of Pain [75] and the Integrated Contextual-Functional Framework [76]. Third, due to the small between-groups effect size (*d* =  -0.05) in the pilot study, the Incidental Learning Task will not be included (and which will provide time for assessments of attentional and memory bias). Fourth, in addition to ambiguous hot sensations, an assessment of interpretation of ambiguous cold sensations will also be conducted, as research suggests people are more sensitive to cold stimuli than hot stimuli [77,78], perhaps due to a greater overall density of cutaneous cold thermoreceptors than warm receptors [79]. Fifth, research has suggested intolerance of uncertainty is associated with higher experimental pain intensity ratings [80]. The Intolerance of Uncertainty Scale-12 Item [81] measure will therefore be added to the questionnaire battery in order to explore associations between intolerance of uncertainty and interpretation of ambiguous somatosensory sensations.

## Protocol for main experimental study

The protocol for the main experimental study is detailed here. Methods and procedures that remain identical to the pilot study will be outlined briefly, while all modifications will be discussed in detail.

### Aims and hypotheses

The primary aim of the main experimental study is to investigate the effects of pain-related CBM-I on the interpretation of ambiguous somatosensory stimuli in healthy, pain-free individuals. The secondary aim is to investigate the effects of CBM-I on patterns of pain-related interpretation, attentional and memory bias. A between-groups design will be implemented, randomising healthy participants to benign CBM-I or pain CBM-I conditions. It is hypothesised that:

1. Participants in the pain modification group will interpret somatosensory stimuli at levels around the pain threshold as more intense and unpleasant than participants in the benign modification group.
2. Participants in the pain modification group will have higher levels of pain-related interpretation bias for ambiguous words and scenarios, as compared to the benign modification group. This is operationalised by:
  a. In the Ambiguous Scenarios Task, participants in the pain modification group will be more likely to interpret ambiguous scenarios as pain-related, as compared to participants in the benign modification group.
  b. In the Sentence Generation Task, participants in the pain modification group will generate more pain-related sentences than participants in the benign modification group.
3. Participants in the pain modification group will have higher levels of pain-related attentional and memory biases bias than participants in the benign modification group.

### Primary and secondary outcomes

The primary outcomes in this study are pain intensity and unpleasantness for ambiguous somatosensory sensations as assessed via the ISSA paradigm. Secondary outcomes are (i) pain-related interpretation biases for ambiguous words and scenarios as assessed via the Ambiguous Scenarios Task and Sentence Generation Task respectively, (ii) pain-related attentional and memory biases as assessed via the visual-probe task and surprise free-recall task respectively, and (iii) anxiety (State-Trait Anxiety Inventory), depression (Beck Depression Inventory-II), anxiety sensitivity (Anxiety Sensitivity Index-3), somatosensory amplification (Somatosensory Amplification Scale), pain catastrophising (Pain Catastrophising Scale), intolerance of uncertainty (Intolerance of Uncertainty Scale-12 Item), and handedness (Edinburgh Handedness Questionnaire – Short Form).

### Participants

Ethical approval has been granted by University of Southampton Research Ethics Committee (ERGO ID: 92317). Eligibility criteria for inclusion are: (i) aged 18–50 years; (ii) normal or corrected to normal vision; and (iii) fluency in the English language. Exclusion criteria are: (i) diagnosis of any type of chronic pain or psychiatric condition; (ii) currently experiencing pain; or (iii) diagnosis or knowledge of any reading difficulties. Participants will be recruited using social media, word-of-mouth, and the website SONA which is used to recruit undergraduate psychology students at the University of Southampton. BSc Psychology students at the University of Southampton will receive partial course credit for participating. A power analysis was conducted using G*Power [82] to determine suitable sample size for an ANOVA, with a medium effect size based on the pilot study which revealed many of the intensity and unpleasantness ratings between benign and pain modification groups were of medium to large effect (effect size *f* =  0.244, α error probability =  0.05, power (1 – *β* error probability) =  0.80, numerator degrees of freedom =  1, and number of groups =  2). This indicates a sample size of 134 is required for these conditions to be met. The start date for this study is 1^st^ June 2024 and the planned end date as registered with the University of Southampton Research Ethics Committee is 1^st^ May 2026.

### Self-report measures

The following self-report measures will be administered in a newly randomised order for each participant: Anxiety Sensitivity Index-3 (ASI-III) [23]; Somatosensory Amplification Scale (SSAS) [32]; Fear of Pain Questionnaire– III (FoP-III) [34]; Pain Catastrophising Scale (PCS) [37]; Beck Depression Inventory-II (BDI-II) (Beck, Steer, & Carbin, 1988); Intolerance of Uncertainty Scale-12 Item (IUS-12) [81]; State-Trait Anxiety Inventory (STAI) [20]; Edinburgh Handedness Questionnaire – Short Form [40]. A demographic questionnaire will also be administered assessing sex, occupation, level of education, personal pain experiences (including number of headache days per month, for females their current phase of menstrual cycle) and prevalence of chronic pain among close relatives. The IUS-12 [81] is new for this main experimental study. This scale includes 12 items assessing an individual’s inclination to find uncertain situations unpleasant. Items are rated on a five-point scale ranging from 1 (Not at all characteristic of me) to 5 (Entirely characteristic of me). Total scores range from 12 to 60, with higher scores indicating greater intolerance of uncertainty. The IUS-12 has demonstrated excellent internal consistency (.91) [81] and acceptable test-retest reliability ((*r* = .77) [83]). Overall, the questionnaires will take approximately 25 minutes to complete.

### Experimental paradigms

Identical to the pilot study, CBM-I training will be achieved via the Ambiguous Scenarios Task [46] modified for pain research by Jones and Sharpe [10]. Assessments of interpretation bias will be made via the test phase of the Ambiguous Scenarios Task and the Sentence Generation task. New to this main experimental study, the probe-position version of the Visual-Probe Task will be used to assess attentional biases for symbolic linguistic stimuli. Specifically, nine sensory-pain and neutral words pairs matched on length and length and Kucera–Francis written frequency [84] will be included (unique to this paradigm and not included in the Sentence Generation Task), which have been validated and used in our former research [62] (Table 4). The task will begin with eight practice trials featuring random letter strings as stimuli. This will be followed by a single block of 64 experimental trials, each of which features one sensory-pain and one neutral word pair. Each trial will begin with a fixation cross in the centre of the screen for 500ms, followed by a randomly selected word-pair presented vertically (i.e., one above the initial fixation cross, the other below) for either 500ms or 1250ms. Immediately after the disappearance of the word-pair, a visual-probe will be randomly displayed in either the upper or lower location replacing one of the former words. Participants must indicate the location of this probe as quickly as possible, using a two-button response-box (with ‘U’ and ‘L’ labels for upper and lower respectively) to provide their response. Following a randomly determined inter-trial interval of either 1000 or 1500 ms, the next trial will begin with the display of the initial fixation cross. The two stimuli presentation times will be applied in a randomised order over all trials. Each of the eight word-pairs will be presented eight times; four times for 500 ms and four times for 1250 ms. Within each exposure duration, each sensory-pain word will appear twice in the upper location and twice in the lower location. The probe location (upper or lower) will be counterbalanced across both locations, resulting in an equal number of congruent (probe replacing the sensory-pain word) and incongruent (probe replacing the neutral word) trials. This task will take approximately 15 minutes to complete. Also new to this main experimental study, a pen and paper version of the free recall task will be used to explore memory biases. Participants will unexpectedly be given two minutes to write down as many words as they can remember from the sentence generation and visual-probe tasks.

**Table 4:** Linguistic stimuli to be used in the visual-probe task.

Sensory-pain words	Neutral words
Throbbing	Autograph
Hurting	Surname
Stabbing	Creation
Painful	Stadium
Harmful	Auction
Aching	Volley
Uncomfortable	Refrigeration
Sore	Echo

### Quantitative sensory testing

#### Pain threshold measurement.

A similar procedure to the pilot study will be followed to assess each participant’s individual pain threshold, although in additional to heat pain thresholds cold pain thresholds will also be assessed the TSA-II previously described For both hot and cold trials the thermal stimulus will increase by 1°C/second from its baseline temperature of 32°C until the participant indicates the very first moment they feel the temperature as painful by pressing the left mouse button. The thermal stimulus has upper and lower safety limits of 53°C and 0°C. The thermal stimulus decreases by 8°C/second back to baseline temperature. Three hot and three cold trials will be conducted, with a 10-second inter-trial interval. The pain threshold measurement will take approximately 10 minutes.

#### Interpretation of somatosensory sensations assessment.

In addition to ambiguous heat stimuli discussed previously, the main experimental study will also assess interpretation of ambiguous cold stimuli administered to the non-dominant hand using the TSA-II. Specifically, cold stimuli at 60%, 80%, 100%, 120% and 140% of an individual’s cold pain threshold will be presented three times each, in a random order set across participants. Percentage threshold temperature values exceeding the safety limits of the TSA-II will be capped at 0°C (i.e., the TSA-II’s safety limits). Each cold trial will begin with the thermal stimulus decreasing from baseline temperature at a rate of 1°C/second until reaching the target temperature. The target temperature will be held for three seconds before returning to baseline at a rate of 8°C/second. At this point, participants will rate via pen and paper visual analogue scales the intensity (from 0 no sensation to 100 most intense pain you can imagine), and the unpleasantness (from 0 not unpleasant at all to 100 extremely unpleasant) of the stimulus. The fifteen cold trials will be presented consecutively with a 10 second inter-trial interval. The order of heat and cold pain trials will be randomised across participants, with the thermode removed for three minutes between each block of trials. This task will take approximately 20 minutes to complete

### Procedure

The procedure for this study is shown in Fig 4. Participant ID numbers will be randomly assigned to the benign or pain modification group before data collection begins. After having read the information sheet and signed the consent form, participants will first complete the questionnaire battery. Following this, participants will complete the pain-threshold measurement task to assess their hot and cold pain thresholds. Next, participants will complete the Ambiguous Scenarios Task aiming to modify their biases towards pain-related or benign interpretations of ambiguous stimuli, according to their randomised group. Immediately following this, participants will complete the Interpretation of Ambiguous Sensation Task. All participants will then complete the battery of cognitive bias tasks. The test phase of the Ambiguous Scenarios Task will be completed first. The order of the sentence generation task and visual-probe task will be randomised and counterbalanced across participants, with the free recall memory bias task conducted last. Lastly, those randomised to the pain modification condition will complete the bias repair task. Participants will complete the study in a single session lasting approximately two hours minutes. Following completion of the study, participants will be thanked and debriefed. The entire experiment will take approximately 120 minutes to complete.

### Data reduction and analytic plan

Data will be analysed using SPSS for Windows 29. For all analyses the significance level will be set to *p* = .05 and two-tailed. Standardized procedures will be followed for identifying and handling missing data and outliers [85]. Visual inspection and descriptive statistics will be performed to identify missing data, and should they exist missing data analysis performed. If <  10% of data is missing and the data is missing at random or completely at random, multiple imputation by chained equations will be used [86,87]. Datasets will be inspected for outliers using box and whisker plots inspect for univariate outliers, and Mahalanobis distance (*D*^*2*^) to detect multivariate outliers [85]. Should outliers exist, their cause will first be established, and if resulting from unique cases or data anomaly, will either be removed or transformed (e.g., log transformation, square root transformation) as appropriate prior to final analysis of the dataset [85].

Descriptive statistics will be computed for demographic characteristics, self-report measures (scored according to their instructions), cognitive bias task scores (Ambiguous Scenarios Task test phase, Sentence Generation Task, Visual-Probe Task and free recall task) and responses on the Interpretation of Ambiguous Somatosensory Sensations Task. Differences between the benign and pain modification groups will be conducted using independent samples *t*-tests and mixed-design ANOVAs where appropriate. Should any statistically significant differences be observed between groups on demographic or self-report measures, analyses will be conducted controlling for these (e.g., Analysis of Covariance). Pearson’s correlation coefficients will be conducted between outcomes on the self-report measures, cognitive bias scores and interpretation of ambiguous sensations ratings. As thermal pain sensitivity is associated with age (e.g., [88–90]), Pearson’s correlation coefficients will also be computed between age and interpretation of ambiguous sensations ratings. Should these be statistically significant, then age will be controlled for as a covariate where appropriate in the statistical analyses. The moderating effects of visual imagery and intolerance of uncertainty on the association between group and interpretation bias scores from the Ambiguous Scenarios Task will be investigated using linear multiple regression models. Similarly, linear multiple regression models will be used to test the effect of group on interpretation bias measures while controlling for other variables that differ between groups.

Data from the Ambiguous Scenarios Task, Sentence Generation Task and ISSA will be processed as discussed above for the pilot study. The only difference is that for the Sentence Generation Task, two raters will independently and blindly categorise participant response sentences as either pain-related (e.g., *He had a pressing pain in his head*) or benign (e.g., *The boy was pressing the buttons in the lift*). The initial inter-rater agreement will be computed, and any disagreements between raters will be discussed in order to reach consensus (involving a third independent member of the research team if necessary).

For the Visual-Probe Task, practice and experimental trials with incorrect responses will be excluded from the analyses. Box and whisker plots for overall data will be inspected to identify response time outliers which will be removed. Mean response times for each participant will then be calculated, with any response time three standard deviations above or below this mean also removed as outliers. Attentional bias scores will then be calculated for each participant at each exposure duration (i.e., 500ms and 1250ms) using the following formula:


$$\text{Attentional bias score=}\left(\left(\text{TuPl – TlPl}\right)\text{+}\left(\text{TlPu – TuPu}\right)\right)\text{/2.}$$

Where T =  threatening stimulus, P =  probe, u =  upper position, l =  lower position.

A positive bias score indicates a shift of attention towards the location of threatening words relative to neutral words. A negative bias score indicates a shift of attention away from the location of threatening words towards neutral words [62]. For the free recall memory bias task, the proportion of accurate words recalled per stimuli category will be computed and used in the analyses [44,70,91].

## Discussion

This paper describes a novel protocol for investigating the effect of pain-related CBM-I on the interpretation of ambiguous somatosensory stimuli. Since the proposal of the concept of the zone of uncertainty around experimental pain thresholds in 2019 [12], no paradigm has been developed to explore this concept within the pain-related bias field. To address this gap, we developed the Interpretation of Somatosensory Sensations Assessment (ISSA), which presents stimuli at 60%, 80%, 100%, 120% and 140% of an individual’s thermal pain threshold and collects ratings on pain intensity and unpleasantness. The results of a small pilot study showed this was suitable for use in a full-powered investigation. No ethical concerns were raised in the use of the ISSA by either the participants or the researchers, and the instructions were clear to participants. The app which calculates heat and cold trial temperatures from the individual participant’s pain threshold is publicly available (see URL above) and can be used by other researchers. Although the ISSA is shown to work well for the purposes of our planned investigation, further testing and development of this paradigm is nevertheless encouraged. For example, while we currently use 20% increments above and below the individual pain threshold, different increments (such as 5% or 10%), if detectable, may create varying levels of ambiguity and should be explored in future research. Furthermore, researchers may wish to explore alternative forms of experimental pain induction such as pressure pain administered via automated pressure cuff algometer, which may more closely resemble pain experienced during clinical procedures (i.e., blood pressure readings).

Overall, the intended aims of the pilot study were met, providing important insights allowing us to make modifications to the experimental design in preparation for the main study. Additionally, our research team (e.g., [3,44,45]) and others (e.g., [75,92–94]) are increasingly calling for research exploring the interrelationships between different forms of cognitive bias. The combined cognitive bias hypothesis proposes that different forms of cognitive bias influence and interact with each other, and these interactions may have a greater impact on maintaining emotional disorders than individual biases in isolation [95]. Specifically for pain, the Integrated Contextual-Functional Framework [76] posits that pain-related biases in attention, interpretation, and memory arise from shared contextual and motivational factors. These biases are likely interconnected and may amplify each other. The protocol detailed here will not only allow us to investigate the effects of pain-related CBM-I on the interpretation of ambiguous somatosensory stimuli, but will also allow us to explore the interrelationships between attentional, interpretation and memory biases for symbolic pain-related stimuli. The results of this research with pain-free individuals will likely inform future research on the transition from pain-free states to acute pain, and subsequently to chronic pain in some individuals. Additionally, the novel ISSA paradigm we developed can be used to further explore the zone of uncertainty across other patient populations who may interpret or evaluate ambiguous bodily sensations differently compared to healthy individuals.

## Supporting information

S1 File Ambiguous scenarios task stimuli. (DOCX)
