# Supplementary material for: A protocol for an experimental investigation of the effects of pain-related interpretation bias modification on interpretation of ambiguous somatosensory and linguistic stimuli in healthy individuals
Source: PLoS One. 2025 Apr 7;20(4):e0318296. doi: 10.1371/journal.pone.0318296 (PMC11975146; doi:10.1371/journal.pone.0318296)
Supplement: S1 File — (DOCX) [file pone.0318296.s001.docx]

**Supplementary Material 1 – Ambiguous Scenarios Task Stimuli**

**Training Phase**

| Ambiguous Scenario | Benign Solution | Pain Solution |
| --- | --- | --- |
| You have been bent over a bucket all morning and are feeling very low on energy. This is because have been….  Context Question:  Were you bent over a bucket because of an illness? (Yes/No) | [cleaning]  cl__n__g | [vomiting]  v_m_t__g |
| Your find that your eyes are sore and swollen. They are so puffy you can barely open them. This is because you are…  Context Question:  Did being allergic cause your eyes to swell? (Yes/No) | [crying]  cr_i_g | [allergic]  all__g_c |
| You’re feeling dizzy, and your head is pounding with heat. You’ re vaguely aware of noises in the background. This is because you are …  Context Question:  Was your dizziness brought on by the sun? (Yes/No) | [sunbathing]  s_n - b_th_ng | [fainting]  fa_nt__g |
| You are at a ceremony for your best friend. All her family and friends are there, and her parents are crying. Your best friend has…  Context Question:  Are you at your best friend’s funeral? (Yes/No) | [married]  m_rr__d | [died]  d__d |
| You are trying to explain yourself, but while this is happening your throat feels increasingly sore and constricted. This is due to…  Context Question:  Is your throat closed up because of an infection? (Yes/No) | [emotion]  em_t__n | [infection]  inf__t__n |
| You see your grandma and she tells you she has an appointment with the doctor to look at two spots on her hand. The spots are caused by…  Context Question:  Has your grandma just been diagnosed with cancer? (Yes/No) | [age]  a_e | [cancer]  c_nc_r |
| You catch up with an old friend, but to your surprise, he is completely bald when you see him. This is because he has…  Context Question:  Is your friend bald only because he shaved his head? (Yes/No) | [shaved]  sh_v_d | [cancer]  c_nc_r |
| You are driving home on the highway, and without realising you crash into another car. It looks pretty bad. You go over to the car to find that the driver is…  Context Question:  Was the driver bleeding? (Yes/ No) | [angry]  a_g_y | [bleeding]  bl__d__g |
| You’re walking in a forest. Suddenly, you trip over and fall onto your knees. Your knees feel all wet, and you look down to see…  Context Question:  Have you come out of the fall without any cuts? (Yes/ No) | [mud]  le_v_s | [blood]  bl__d |
| You’re in a restaurant. On a table nearby you see a man gasping for air. His body is shaking and his mouth is wide open. He is…  Context Question  Was the man in the restaurant laughing? (Yes/No) | [laughing]  la_g__ng | [choking]  ch_k__g |
| You take your place in the line. On a small table in front of you there are a few magazines. You notice that there are still two people in the queue in front of you. It is annoying that you have to wait this long for the…  Context Question:  Were you waiting in the doctor’s waiting room? (Yes/No) | [hair dresser]  ha_r - dr_ss_r | [doctor]  do_t_r |
| It’s dark outside but you just can’t sleep. You are very aware of the people around you who are keeping you up with their coughing. You are in a…  Context Question:  Did the people in the hospital keep you up at night? (Yes/ No) | [hostel]  h_st_l | [hospital]  ho_p__al |
| Beads of sweat are covering your forehead. Your heart is pounding very hard and your breathing is irregular. This is because you are…  Context Question:  Is your heart pounding because you have been jogging? (Yes/No) | [jogging]  j_gg_ng | *having a* [heart-attack]  he__t - at__ck |
| You and your friend are preparing dinner together. She cuts the onion and you cut the pepper. Suddenly, her knife slips, and cuts into your…  Context Question:  Did you cut yourself? (Yes/No) | [pepper]  pe_p_r | [finger]  fi_g_r |
| The woman walks slowly towards you. She seems to be making every effort not to lose her balance. It’s hard for her to walk with…  Context Question  Was the woman struggling to walk because of high-heels? (Yes/No) | [high-heels]  h__gh - h__ls | [crutches]  cr_t__es |
| You’re walking on the street. You see a man a few meters in front slip and fall hard onto the pavement. When you get to him, he is lying in a large puddle of…  Context Question:  Was the man lying in his own blood? (Yes/No) | [water]  w_t_r | [blood]  bl__d |
| You are chatting with an experienced skier. He took part in major international ski competitions. Not so long ago he broke a…  Context Question:  Did the skier injure himself recently? (Yes/No) | [record]  r_c_rd | [leg]  l_g |
| The man sitting next to you suddenly jumped right out of his seat, and gripped both hands immediately to his chest and face. He was…  Context Question:  Was the man next to you sick? (Yes/No) | [shocked]  sh_ck_d | [sick]  s__k |
| Your father had cancer. You had to go back every month with him to the hospital for treatment, but that now that no longer happens. This is because he recently…  Context Question:  Did your father recently pass away? (Yes/No) | [recovered]  r_co__red | [died]  d__d |
| You go to visit your grandmother in the nursing home. You find her lying in her bed, facing upwards and hardly moving. She is…  Context Question:  Was your grandmother dead when you found her? (Yes/No) | [sleeping]  sl__p__g | [dead]  d__d |
| You are gasping for air, and seem unable to fill your lungs enough. Your chest is moving up and down very quickly, and it’s not getting any easier to breath. You are…  Context Question:  Are you asthmatic? (Yes/No) | [working-out]  wo_k__g - o_t | [asthmatic]  as__ma__c |
| The operation was carefully planned, but regardless of this, the end result was a disaster. Consequently, the young man had to be taken away by the…  Context Question:  Was the young man taken by the paramedics? (Yes/No) | [police]  po__ce | [paramedics]  par_m_d_cs |
| You have a bulge on your foot, and as a result, it is difficult to get your shoe on. You went to the doctors and they told you it was a…  Context Question:  Do you have a tumour? (Yes/No) | [bite]  b_te | [tumour]  t_m__r |
| On the other side of the street is a woman who is struggling with the uneven pavement. She has to use a lot of force to push her…  Context Question:  Did the woman struggle with her wheelchair? (Yes/No) | [pram]  pr_m | [wheelchair]  wh__lch__r |
| Your skin is hot and irritated to the point where it is now glowing red. The alarm for the machine goes off. It is now time to stop with the…  Context Question:  Are you undergoing radiation treatment? (Yes/No) | [sunbed]  s_n-b_d | [radiation]  r_di_t__n |
| Yesterday, a vehicle stuck your bike. Now you cannot ride because at the time of the fall you broke your…  Context Question:  Is your leg now broken as a result of the crash? (Yes/No) | [bike]  b__e | [leg]  l_g |
| You walk across the road because you spot your friend Kim. She has lost a lot of weight and looks skinny. This is the result of…  Context Question:  Does your friend Kim have a disease? (Yes/No) | [diet]  d__t | [disease]  di___se |
| You tried to warn Jack, but it was already too late. He fell over and stained the carpet with…  Context Question:  Did Jack stain the carpet with his blood? (Yes/ No) | [wine]  wi_e | [blood]  bl__d |
| You’re on a holiday in Brazil and a mosquito stings you. You are worried, and decide to go to the doctor. He tells you that you have caught…  Context Question:  Did the mosquito cause you to catch Malaria? (Yes/ No) | [nothing]  n_th__g | [malaria]  ma__r_a |
| You have a shower and then finish. Once you finish drying your hair, you hang your towel up and notice it’s covered in red patches. The red patches are…  Context Question:  Did you find blood on your towel? (Yes/No) | [hair-dye]  h__r - d_e | [blood]  bl__d |

**Test Phase**

| Descriptive Scenario | Sentence Type | Sentences |
| --- | --- | --- |
| *The Thriller Movie*    You are watching a thriller movie where the killer is currently hunting down the main character. The main character walks slowly around the corner to find herself face to face with the killer, and they start to sc_e_m  [scream]  Were you watching a thriller movie? (Yes/No) | Benign Target | As the main character turns the corner, the killer tries to grab her, but she runs away |
|  | Negative Target | As the main character turns the corner, she sees the killer and is stabbed and cries out in pain |
|  | Benign Foil | As the main character turns the corner she sees the killer is someone she knows |
|  | Negative Foil | As the main character turns the corner, she sees the killer with a knife |
| *The Water Park*    You and your friends decide to go to the water slide park while on your holiday. It was a really fun day, and you are all exhausted. One of your friends forgot to bring a change of clothes and is still wet, and as a result she is still now sh_v_r_ng  [shivering]  Did you go to a water park with your friends (Yes/No) | Benign Target | Your friend was shivering, but once you got out of the cold she got warm |
|  | Negative Target | Your friend was shivering, and because of her wet clothes she developed a cold |
|  | Benign Foil | Your friend was shivering, and when you got home her clothes were still wet |
|  | Negative Foil | Your friend was shivering, and her wet clothes were very uncomfortable |
| *The Iphone Accident*    You are walking across a street in the middle of writing a text to a friend. Because you are not looking where you are going, you bump into a p_le [pole]  Were you texting a friend whilst walking (Yes/No) | Benign Target | You bumped into a pole, but you’re not hurt and no one saw |
|  | Negative Target | You bumped into a pole with your face and are now bleeding |
|  | Benign Foil | You bumped into a pole and a car nearby honked at you |
|  | Negative Foil | You bumped into a pole, and you were embarrassed |
| *The Kitchen*  You and your brother are fighting in the kitchen. He says that you cannot understand where he is coming from. In his frustration, he slams the fridge d__r [door]  Did you brother slam the fridge door (Yes/No) | Benign Target | He slammed the fridge door and walked away angrily |
|  | Negative Target | He slammed the fridge door and as a consequence, jammed your finger in there |
|  | Benign Foil | He slammed the fridge door and went silent |
|  | Negative Foil | He slammed the fridge door and continued to shout at you |
| *The Doctors*    Your mum has been worried about a lump in her breast for some time now. 2 weeks ago she went to get a mammogram, and you are now in with her talking to the doctor. The doctor politely asks you to leave the r__m [room]  Did the doctor request that you leave him and your mum alone? (Yes/No) | Benign Target | The doctor asked you to leave due to patient confidentiality |
|  | Negative Target | The doctor asked you to leave because he is telling your mum she has cancer |
|  | Benign Foil | The doctor asked you to leave because you were distracting |
|  | Negative Foil | The doctor asked you to leave because you may not want to hear what he has to say |
| *At the Gym*    You are doing your weekly work-out at the gym. The guy next to you is really pushing himself, and is panting like crazy. Within minutes of you being there, you hear a loud so_nd [sound]  Were you working out at the gym? (Yes/No) | Benign Target | The loud sound was the man celebrating that he has finished his work-out |
|  | Negative Target | The loud sound was the man yelling because he had dropped the dumbbell on his toe |
|  | Benign Foil | The loud sound was someone moving a heavy weight |
|  | Negative Foil | The loud sound was him shouting because he is exhausted |
| *The Pharmacy*    Your hands are dry and itchy. You decide to go to the pharmacist, and amongst the bag of things you have bought is a cr__m [cream]  Were your hands itchy and dry? (Yes/No) | Benign Target | The cream you bought is a shaving cream |
|  | Negative Target | The cream you bought is a medicated, to soothe the pain from eczema |
|  | Benign Foil | The cream you bought is a daily moisturizer for your face |
|  | Negative Foil | The cream you bought is a moisturizer to soothe your itchy skin |
| *The Baguette*    You are walking along a busy street eating a baguette for lunch with lots of filling. It’s quite hard to eat, but delicious all the same. You take a new mouth full, and have to stop ch_w_ng [chewing]  Were you eating a baguette on a busy street? (Yes/No) | Benign Target | You stopped chewing because you bumped into someone by accident |
|  | Negative Target | You stopped chewing because you bit your tongue in the process of eating |
|  | Benign Foil | You stopped chewing because something caught your eye |
|  | Negative Foil | You stopped chewing because your jaw was sore |
| *Moving House*    You are moving house today, and boxes of all your belongings surround you. Your friend Jack is helping you for the day. You walk into another room, and hear a rather loud b_ng [bang]  Was your friend Jack helping you move house? (Yes/No) | Benign Target | The bang was Jack knocking something over as he walked past |
|  | Negative Target | The bang was Jack dropping a box because he pulled a muscle in his back |
|  | Benign Foil | The bang was a door slamming because of the wind |
|  | Negative Foil | The bang was Jack tripping over and falling |
| *The Road Trip*    You and your friend are on a road tip going North. You have the music playing loudly, and have plenty of snacks to eat on the journey. Everything is fun until a car in the left lane swerves sharply and causes you to have a cr_sh [crash]  Were you and your friend on a road trip? (Yes/No) | Benign Target | The crash wasn’t serious as you and your friend are both okay |
|  | Negative Target | The crash was pretty bad; your friend has whiplash from the sudden braking |
|  | Benign Foil | The crash wasn’t serious though the car will definitely need fixing |
|  | Negative Foil | The crash was pretty bad, you’re not sure if the other driver is okay |
